# Hollowing out MOFs: hierarchical micro- and mesoporous MOFs with tailorable porosity *via* selective acid etching†
†This work was supported by the Institute for Basic Science (IBS) [IBS-R007-D1].
‡
‡Electronic supplementary information (ESI) available. See DOI: 10.1039/c7sc02886e



**DOI:** 10.1039/c7sc02886e

**Published:** 2017-08-09

**Authors:** Jaehyoung Koo, In-Chul Hwang, Xiujun Yu, Subhadeep Saha, Yonghwi Kim, Kimoon Kim

**Affiliations:** a Center for Self-assembly and Complexity (CSC) , Institute of Basic Science (IBS) , Pohang , 37673 , Republic of Korea . Email: kkim@postech.ac.kr ; http://csc.ibs.re.kr; b Department of Chemistry , Pohang University of Science and Technology , Pohang , 37673 , Republic of Korea

## Abstract

We report a new strategy for the synthesis of robust hierarchical micro- and mesoporous MOFs from water stable MOFs *via* a selective acid etching process. The process is controlled by the size-selective diffusion of acid molecules through the MOF windows. This method enables the fine-tuning of the porosity of hierarchical MOFs, allowing for the generation of well-defined mesopores with high mesopore volume. Because of the size-selective diffusion of acid molecules, the inherent crystallinity and external morphology of the resulting MOFs are well-maintained after acid treatment. This novel strategy may provide an alternative route towards the synthesis of diverse hierarchical MOFs.

## Abstract 2

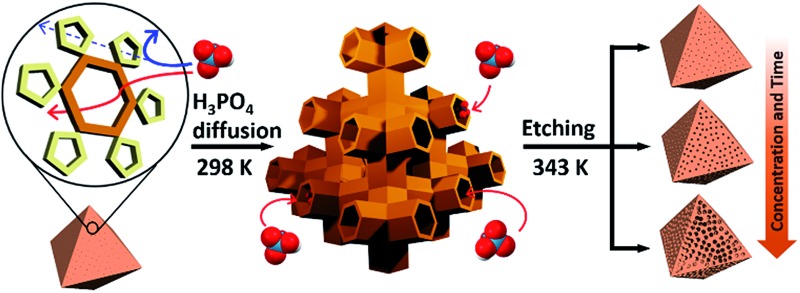
We report a new strategy for the synthesis of robust hierarchical micro- and mesoporous MOFs from water stable MOFs *via* a selective acid etching process.

## Introduction

Metal–organic frameworks (MOFs) are crystalline porous materials constructed *via* the self-assembly of metal ions (or clusters) and organic ligands,^1^ which have widespread applications in many fields owing to their processability, structural flexibility, and well-defined pores with large surface areas.^2^ Such properties are often directly related to the size, geometry and accessibility of the pores.^3^ Microporous MOFs (pore size < 2 nm) possess high surface area with structural selectivity for small molecules, but often have limited applicability because of the difficulties in mass transfer and encapsulation of functional large guest molecules.^4^ To overcome these limitations, mesoporous MOFs (2–50 nm) have become a subject of great interest.^5,6^ However, mesoporous MOFs often collapse upon the evacuation of guest molecules and the pore sizes of the MOFs produced by conventional solvothermal methods are usually smaller than 5 nm.^7,8^

Recently, hierarchical micro- and mesoporous MOFs have emerged as a promising alternative combining the advantages of both micro- and mesoporosity.^9^ For example, mesopores facilitate the mass transfer process and the concomitant micropores offer high surface area. Furthermore, hierarchical micro- and mesoporous MOFs can enhance catalytic activities, and have been utilized as hosts for large guest molecules (*e.g.*, enzymes).^10^ Therefore, several strategies have been developed to construct hierarchical porous MOFs *via* crystal growth as well as post-synthetic procedures, such as imperfect crystallization, gas-expanded liquid, template-assisted, modular induced, and calcination methods.^11^ Although these strategies are novel and inventive, they often involve lengthy synthetic procedures and fine control of the pore sizes with high structural stability *via* a single step procedure remains a significant challenge.

**Scheme 1:**
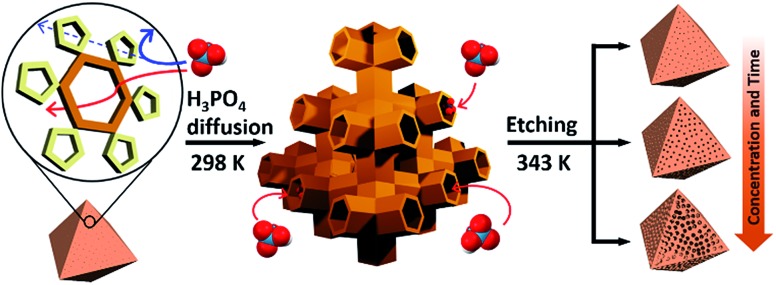
Illustration of the etching process for MIL-100(Fe). Left: MIL-100(Fe) crystal with hexagonal and pentagonal windows; middle: acid diffusion into tetrahedral channels through hexagonal windows; right: resulting mesopores after etching.

Recently, we reported a simple hydrolytic method to synthesize a hierarchical micro- and mesoporous MOF using a microporous MOF (POST-66).^12^ However, this method is only applicable to MOFs with low water stability and the fine-tuning of pore size still needs to be addressed. Here, we report a novel strategy for the synthesis of water-stable hierarchical porous MOFs by a selective acid etching process (Scheme 1). This method not only allows fine tuning of the porosity, but also preservation of the inherent crystallinity and external morphology of the resulting MOFs due to the selectivity of the etching process. Although we demonstrate the principle mainly with MIL-100(Fe) [Fe_3_(μ_3_-O)(H_2_O)_2_(OH)(BTC)_2_], BTC = benzene-1,3,5-tricarboxylate), this strategy can be extended to the synthesis of other water-stable hierarchical porous MOFs.

## Results and discussion

**Fig. 1:**
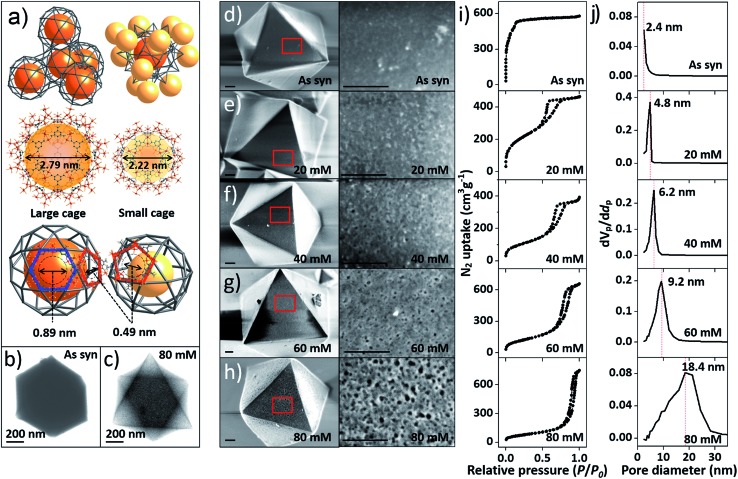
(a) Top-left: tetrahedral geometry constructed by large cages; top-right: a large cage surrounded by 12 adjacent small cages; middle: structures of the large and small cages; bottom: hexagonal and pentagonal windows. (b and c) TEM images of pristine MIL-100(Fe) and MIL-100(Fe)-80. (d–h) SEM images of pristine and acid-etched MIL-100(Fe) (scale bars: 200 nm), (i) N_2_ sorption isotherms (at 77 K) and (j) pore size distribution profiles of as-synthesized MIL-100(Fe) and MIL-100(Fe)-20, 40, 60 and 80. The whole plots are provided in Fig. S7 and S8.‡

At the outset of this work, we thought that we could exploit the window dimensions of MIL-100(Fe) to allow selective acid etching of the MOF due to the fact that MIL-100(Fe) has large and small cages with hexagonal and pentagonal windows (*d* = 0.89 and 0.49 nm, respectively) (Fig. 1a and S1‡).^13^ Therefore, if we employed an appropriately sized inorganic acid as an etching agent, it may diffuse into MIL-100(Fe) through the hexagonal windows but not the pentagonal windows allowing a selective etching process, while retaining the overall crystallinity. Considering these points, we chose phosphoric acid (H_3_PO_4_, *d* = 0.61 nm)^14^ in *N*,*N*-dimethylformamide (DMF) as an etching agent, which exhibits size-selective diffusion into the 3D channels of the large cages through the hexagonal windows at room temperature. The etching process then takes place after raising the temperature (Scheme 1 and Fig. S2‡).

With this idea in mind, we synthesized MIL-100(Fe) following the literature procedure.^13^ Subsequently the dehydrated MIL-100(Fe) powder was soaked in the H_3_PO_4_ solution at room temperature under sonication for enhanced diffusion, and then heated to 70 °C for the etching. The concentration of the acid solution was varied (0 to 80 mM) in order to control the degree of etching.

Transmission electron microscopy (TEM) images (Fig. 1b and c) showed that the acid treated sample (MIL-100(Fe)-80, treated by 80 mM H_3_PO_4_ solution) has a sponge-like morphology and much higher transparency compared to pristine MIL-100(Fe) because of the enlarged pore volume. Scanning electron microscope (SEM) images (Fig. 1d–h) indicated that increasing the concentration of H_3_PO_4_ (0 to 80 mM) resulted in regular enlargement of the pore sizes on the crystal surface while maintaining the morphology of MIL-100(Fe) (crystal size and shape). Also, a series of powder X-ray diffraction (PXRD) profiles (Fig. S4a‡) confirmed the maintenance of the original crystallinity and energy-dispersive X-ray spectroscopy (EDS) analyses showed that the atomic percentage of Fe^3+^ ions was constant (Fig. S5‡). Moreover, there was no change in the oxidation state of the Fe(iii) as shown by the X-ray photoelectron spectroscopy (XPS) spectra (Figure S4b‡).

The porosities of the acid treated MIL-100(Fe) series were investigated by N_2_ sorption measurements. The gradual change of the adsorption–desorption isotherms from microporous type I to mesoporous type IV (Fig. 1i and S7‡) indicates the partial loss of micropores and the generation of mesopores (Fig. S3‡).^15^

Additionally, the sorption isotherm has a H1 type hysteresis loop, which is characteristic of well-defined pore channels.^16^ Owing to the increasing void space, the N_2_ sorption capacity of MIL-100(Fe)-80 reaches up to 750 cm^3^ g^–1^ and the mesopore volume of MIL-100(Fe)-80 is 1.15 cm^3^ g^–1^ (Table S1‡). These values are comparable to that of an ultra-high mesoporous MOF.^10^ The mesopore sizes, calculated from Barrett–Joyner–Halenda (BJH) analysis, gradually increased from 2.4 to 18.4 nm (Fig. 1j and S8‡) and had a narrow pore size distribution with high differential pore volume. Acid concentrations above 70 mM resulted in the broadening of the pore size distribution profiles. Furthermore, the mesopore sizes of the acid-treated MIL-100(Fe) can also be fine-tuned by adjusting the treatment time with a fixed H_3_PO_4_ concentration (Fig. S9‡). With the increase of acid treatment time from 2 to 12 h, the mesopore size gradually increased from 3.3 to 18.4 nm (Fig. S10‡).

During the etching process, Fe^3+^ ions and BTC ligands (constituents of MIL-100(Fe)) gradually leached from the frameworks and the leaching amount of both components was proportional to the acid concentration, as confirmed by inductively coupled plasma-atomic emission spectrometry (ICP-AES) and UV-Vis spectroscopy (Fig. S12‡).

**Fig. 2:**
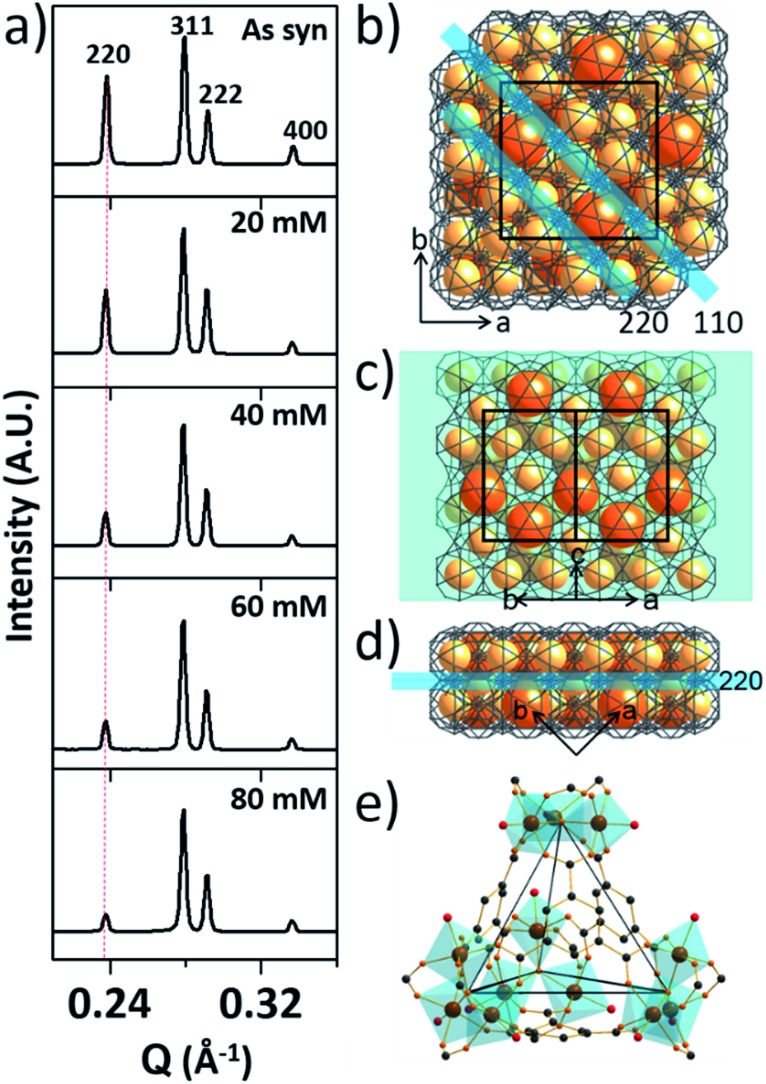
(a) SAXS spectra of the pristine MIL-100(Fe) and MIL-100(Fe)-20, 40, 60 and 80. (b) The unit cell of MIL-100(Fe) with the (110) and (220) lattice planes (blue colored) and (c and d) front (cross section) and side views of the (220) lattice plane. (e) Structure of the tetrahedral secondary building unit (SBU) of MIL-100(Fe).

To investigate the etching process in more detail, the local structures of the hierarchical porous MOFs were studied by small-angle X-ray scattering (SAXS) and high resolution TEM (HR-TEM). The series of acid-treated MIL-100(Fe) samples had identical peaks in the SAXS spectra (Fig. 2a), suggesting retention of the crystallinity. The full width at half maximum (FWHM) of the diffraction peaks, (220), (311), (222) and (400), slightly increased at higher acid concentrations (Fig. S13‡), implying successful pore enlargement,^17^ which is consistent with the power law scattering of Q^–4^ in the SAXS profiles (typical scattering behaviour of mesoporous MOFs, Fig. S14‡).^18^ In particular, the intensity of the (220) peak which corresponds to the lattice plane on the boundary between the large and small cages significantly decreased from MIL-100(Fe) to MIL-100(Fe)-80 (Fig. 2b–d). Due to the size-selective diffusion of H_3_PO_4_, the etching process probably proceeded along the large cages on the (220) plane, causing the significant intensity decrease of the (220) peak. Hence, the secondary building units (SBUs, Fig. 2e) of the large cages on the (220) lattice plane disassembled gradually and the large and small cages merged to form expanded pores.

**Fig. 3:**
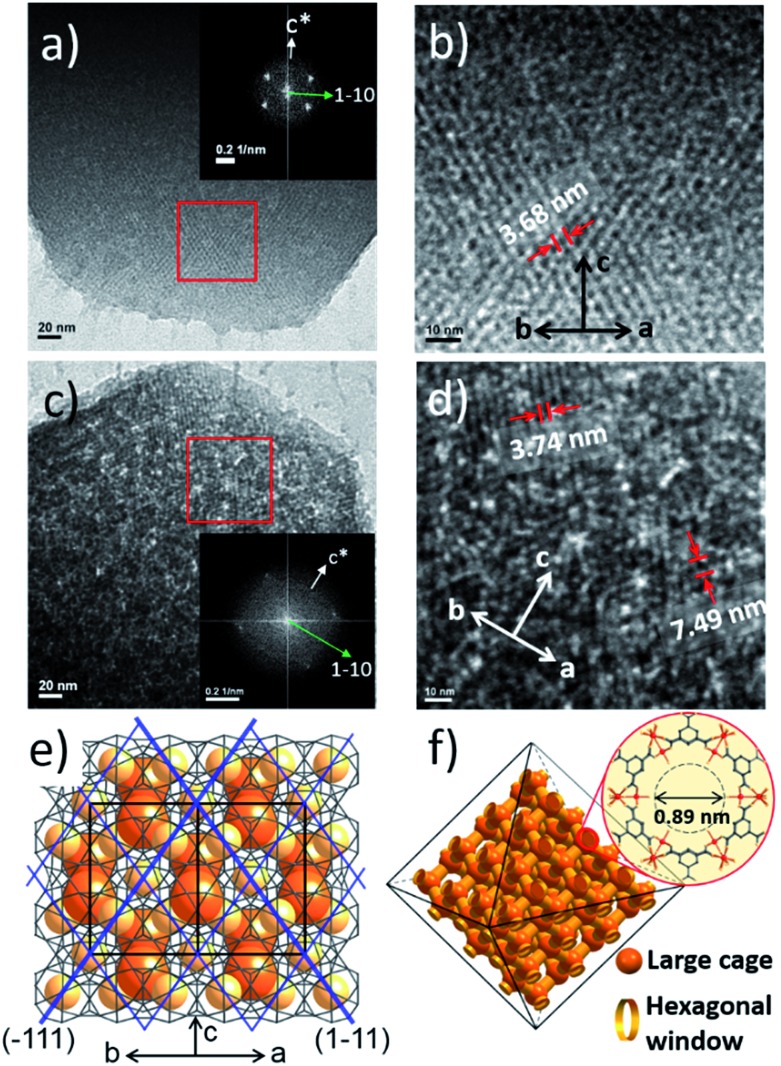
(a–d) TEM images of MIL-100(Fe)-40 (a and b) and 80 (c and d). The insets are the corresponding fast Fourier transform electron diffraction patterns (FFT-EDP) of the red areas. (e) The structure of the (220) lattice plane with a series of (1–11) lattices (blue lines). (f) Tetrahedral 3D channel of the large cages.

Additionally, the HR-TEM images of MIL-100(Fe)-40 and 80 (Fig. 3a–d) with clear lattice fringes of orientation (1–11) (*d* = 3.68 and 3.74 nm, respectively), demonstrated that higher acid concentration resulted in a greater degree of etching. This observation indicates that the crystalline structure is maintained after the etching process and that the mesopore transformation occurred with a preferential direction along the [220] zone axis, in accordance with the SAXS analysis. Moreover, the enlarged lattice fringe orientated along the (1–11) lattice plane (*d* = 7.49 nm, Fig. 3d) corresponds to a doubling of the (1–11) *d*-spacing value (blue lines, Fig. 3e). The enlargement was probably caused by the merged cages derived from etching the SBUs in between the large and small cages on the (220) plane. This clearly supports the idea that the mesopore enlargement process occurred in a structurally selective manner.

Consequently, the observed etching process can be explained as follows (Scheme 1). Due to the size difference between the hexagonal and pentagonal windows, H_3_PO_4_ selectively diffuses into the 3D tetrahedral channels of the large cages through hexagonal windows at room temperature (Scheme 1 and Fig. 3f). After activation (heating at 70 °C), the SBUs of the large cages near the (220), (022) and (202) lattice planes (boundaries between the large and small cages, SAXS, Fig. 2) are gradually etched, resulting in the disassembly of the [(Fe_3_(μ_3_-O))(OH)(H_2_O)_2_]^6–^ and BTC linker (ICP-AES and UV-Vis spectra, Fig. S12‡). This disassembly leads to the merging of the small and large cages, while the majority of the walls of the small cages survive to maintain the crystallinity and the structural integrity. Finally, the local structure collapses and the mesopores are generated (TEM data, Fig. 3).

To further validate the size-selective acid diffusion of the etching process, sulfuric acid (H_2_SO_4_, *d* = 0.66 nm (ref. 19)), which has a comparable size to H_3_PO_4_, and hydrochloric acid (HCl, *d* = 0.34 nm (ref. 20)) were employed as etching agents. In the case of H_2_SO_4_, the mesopore size of hierarchical porous MIL-100(Fe) can also be tuned in a controlled manner (Fig. S15‡). However, hydrochloric acid, because of its smaller molecular dimension, can easily diffuse into both the hexagonal and pentagonal windows (loss of size selectivity), resulting in the collapse of the whole framework of MIL-100(Fe) (dissolution in the acid solution).

Preliminary studies have suggested that this strategy can be applied to other water-stable MOFs which have a structural selectivity for H_3_PO_4_ (*e.g.*, soc-MOF and MIL-88A). As demonstrated by the structure-property data and N_2_ sorption data shown in Fig. S16, S17, S19 and S20,‡ the porosity of soc-MOF and MIL-88A can also be controlled in a range under 20 nm by adjusting the acid concentration. Besides, the crystallinity and morphology is still retained after acid treatment as evidenced by XRD patterns and TEM images (Fig. S18, S21 and 22‡).

## Conclusions

We have developed a general strategy to prepare hierarchical micro- and mesoporous MOFs from water-stable MOFs (MIL-100(Fe), soc-MOF, and MIL-88A). This work demonstrated that: (i) the size-selective acid diffusion strategy is a versatile method to control the etching process; and (ii) control of the acid concentration and the treatment time can produce hierarchical MOFs with the desired pore size dimensions while maintaining the original microporosity and structural stability. This simple strategy may provide an alternative route towards the synthesis of tailor-made hierarchical MOFs and hold enormous promise for facilitating the development of MOF-based materials with interesting properties. Work along this line is currently underway in our laboratory.

## Experimental

### General information

All the reagents and solvents were commercially available and used as supplied without further purification. ICP-AES (IRIS Intrepid II XSP, Thermo Electron Corporation) was used for the analysis of metal ion concentrations. UV-Vis absorption spectra were collected by an Agilent Cary 5000 UV-Vis-NIR Spectrophotometer. TEM, HR-TEM and STEM images were measured using a JEOL JEM-2200FS with image Cs-corrector equipped (National Institute for Nanomaterials Technology (NINT), Korea). SEM images were collected by a JSM 7800F PRIME scanning electron microscope operating at 1 kV. Powder XRD patterns were obtained on a Rigaku Smartlab system equipped with a Cu sealed tube (wave length = 1.54178 Å) and a vacuumed high-temperature stage (Anton Paar TTK-450). The following conditions were used: 40 kV, 30 mA, increment = 0.01°, and scan speed = 0.3 s per step. NMR data were recorded on a Bruker DRX500 spectrometer. Small-angle X-ray scattering (SAXS) measurements were carried out using the 4C SAXS II beamline (BL) of the Pohang Light Source II (PLS II) with 3 GeV power and an X-ray beam wavelength of 0.734 Å at the Pohang University of Science and Technology (POSTECH), Korea. The magnitude of the scattering vector, *q* = (4π/*λ*) sin *θ*, was 0.1 nm^–1^ < *q* < 6.50 nm^–1^, where 2*θ* is the scattering angle and *λ* is the wavelength of the X-ray beam. All scattering measurements were carried out at 25 °C.

### Gas adsorption measurements

All gas sorption isotherms were measured at 77 K with BELSORP-mini volumetric adsorption equipment. Typically, a sample of as-synthesized material (∼100 mg) was loaded and, prior to the measurements, residual solvents were exchanged with EtOH for 3 days, and then evacuated by heating to 200 °C under a high vacuum (10^–2^ Pa).

### Mesoporous transformation procedure of MIL-100(Fe)

MIL-100(Fe) was synthesized following literature procedure.^13^ As-synthesized MIL-100(Fe) was first dehydrated and then soaked in DMF (6 mL) with different amounts of phosphoric acid (89 wt%, TCI, Japan) to give solutions with varied acid concentration. All samples were sonicated for 10 minutes at room temperature and then kept at 70 °C in an oven. For concentration controlled etching, the treatment time was kept constant at 5 hours. While for treatment time controlled etching, the treatment time was varied from 2 to 12 hours in 40 mM H_3_PO_4_. After the acid etching was completed, the crystalline solid materials were washed with DMF and EtOH 3 times each and dried under vacuum overnight.

## Conflicts of interest

There are no conflicts to declare.
