# Supplementary material for: Hollowing out MOFs: hierarchical micro- and mesoporous MOFs with tailorable porosity via selective acid etching
Source: Chem Sci. 2017 Aug 9;8(10):6799–803. doi: 10.1039/c7sc02886e (PMC5643979; doi:10.1039/c7sc02886e)
Supplement: Supplementary file 1 [file SC-008-C7SC02886E-s001.pdf]

Electronic Supplementary Material (ESI) for Chemical Science.
This journal is © The Royal Society of Chemistry 2017

Supporting Information

Hollowing Out MOFs: Hierarchical Micro- and Mesoporous MOFs with Tailorable Porosity via Selective Acid Etching

*Jaehyoung Koo, In-Chul Hwang, Xiujun Yu, Subhadeep Saha, Yonghwi Kim and Kimoon Kim**

Department of Chemistry, Pohang University of Science and Technology (POSTECH), Pohang, 37673, Republic of Korea; Center for Self-assembly and Complexity (CSC), Institute of Basic Science (IBS), Pohang, 37673, Republic of Korea.

Fax: +82 54-279-8129; Tel: +82 54-279-2113; E-mail: kkim@postech.ac.kr

Contents

1. **Supplementary Figures** (S2 - S15)

2. **Supplementary Tables** (S16)

3. **References** (S17)

1. Supplementary Figures

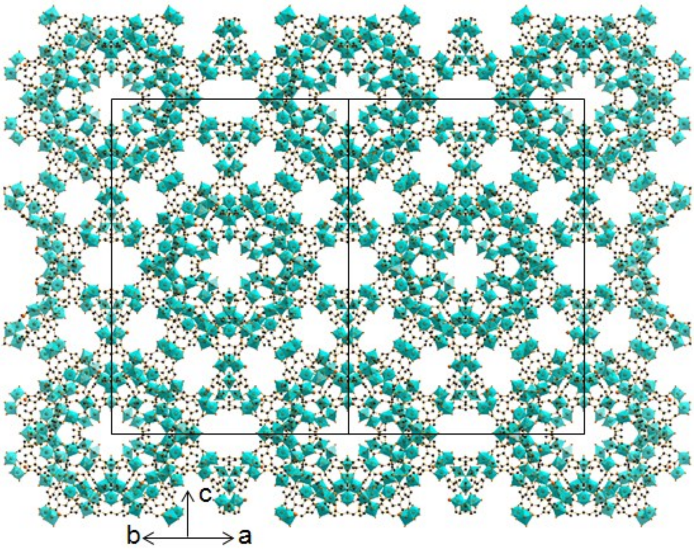

Figure S1. Unit cell structure of MIL-100(Fe)[1] viewed along the (110) plane and octahedrons represent Fe(III) complexes. The unit cell of the MIL-100(Fe) framework consists of 8 large cages (diameter is 2.79 nm) and 16 small cages with a diameter of 2.22 nm (Figure 1b). The large cage is comprised of 4 hexagonal windows (diameter is around 8.85 Å) and 12 pentagonal windows (diameter is around 4.91 Å). On the other hand, the small cage is constructed by 12 pentagonal windows. Each of the large cages are surrounded by 12 small cages and they are connected through the pentagonal windows. Also, the large cage is linked to each other by sharing the hexagonal windows and, as a result, form a tetrahedral geometry, which link together to form a 3D network channel through the hexagonal windows.

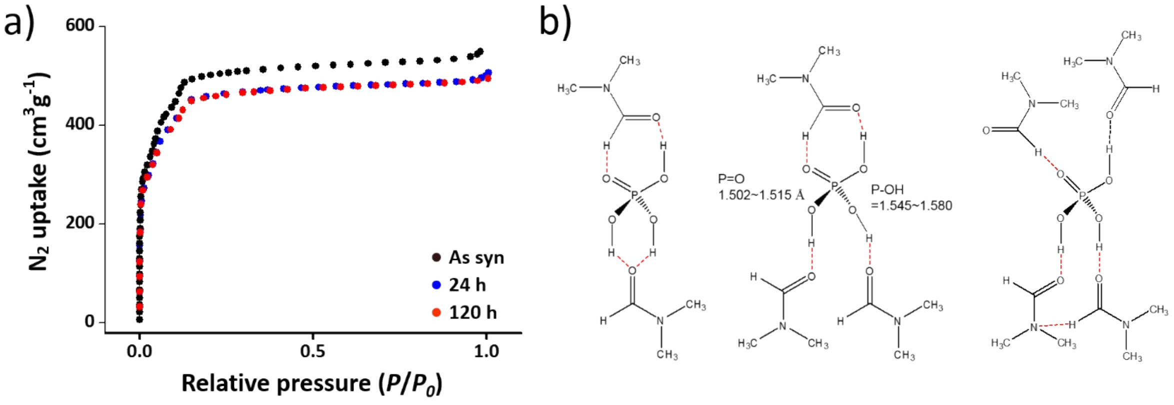

Figure S2. a) N_2 sorption isotherms of MIL-100(Fe) treated with H_3PO_4/DMF at room temperature for 24 and 120 hours. **b**) The models of intermolecular H-bonding in solution of H_3PO_4-DMF, which indicates H_3PO_4 can be stabilized by DMF at room temperature.[2]

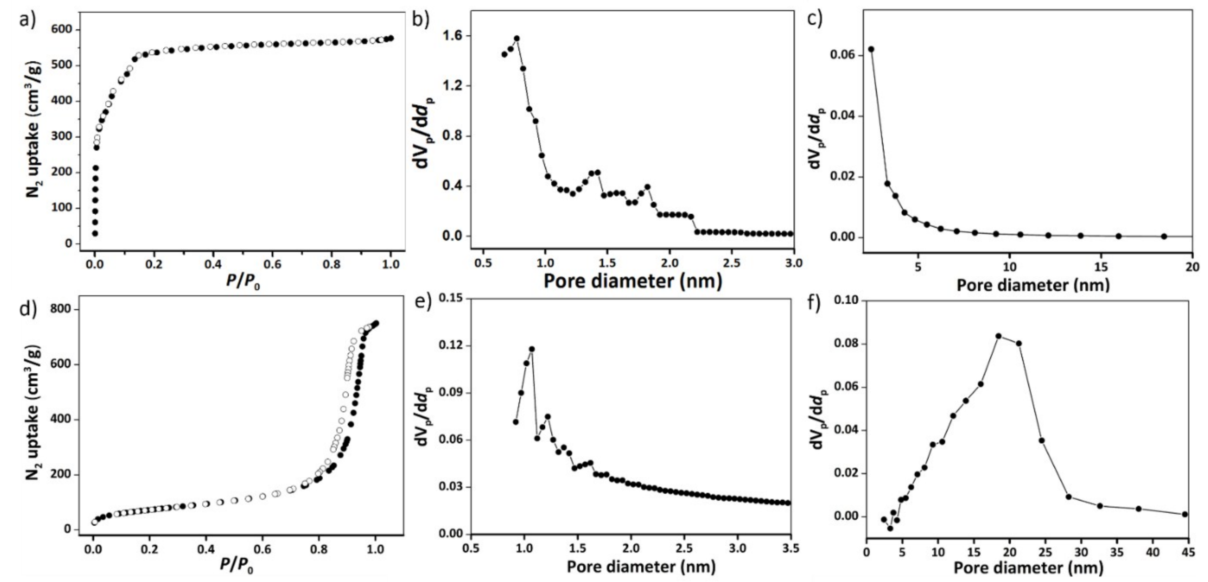

Figure S3. N_2 sorption isotherms (left), micropore distribution (middle) and mesopore distribution (right) of MIL-100(Fe) (a-c) and MIL-100(Fe)-**80** (d-f). The N_2 adsorption data of MIL-100(Fe) show a type I (microporous) isotherm with a secondary uptake before $p/p_0 = 0.1$, which indicates the presence of two kinds of pores.[1] While N_2 adsorption of MIL-100(Fe)-**80** exhibits a type IV isotherm. The pore size distribution was calculated by both Horvath-Kawazoe (HK) for micropore and Barrett-Joyner-Halenda (BJH) for mesopore analytical methods.

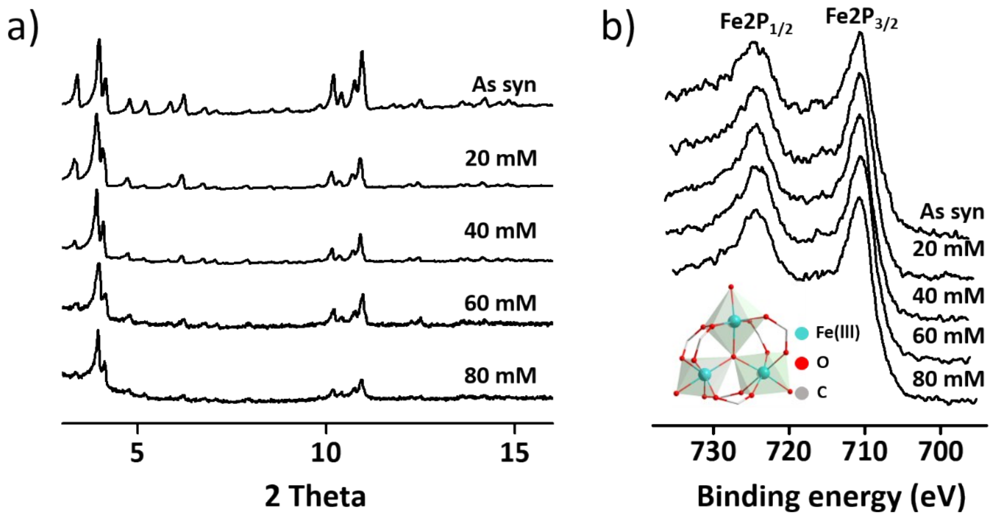

Figure S4. PXRD profiles **a**) and XPS spectra **b**) of as-synthesized MIL-100(Fe) and MIL-100(Fe)-**20**, **40**, **60**, **80**.

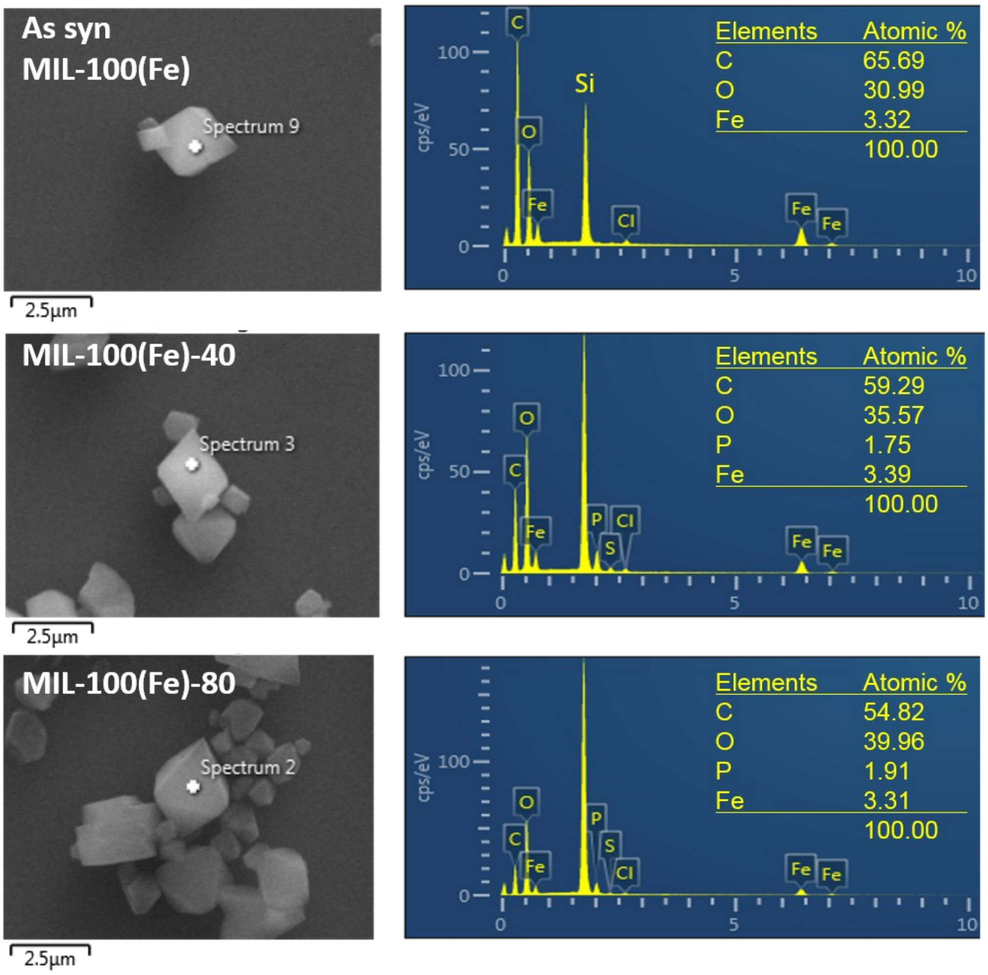

Figure S5. SEM images (left) and EDS profiles (right) of as-prepared MIL-100(Fe), MIL-100(Fe)-**40** and **80**.

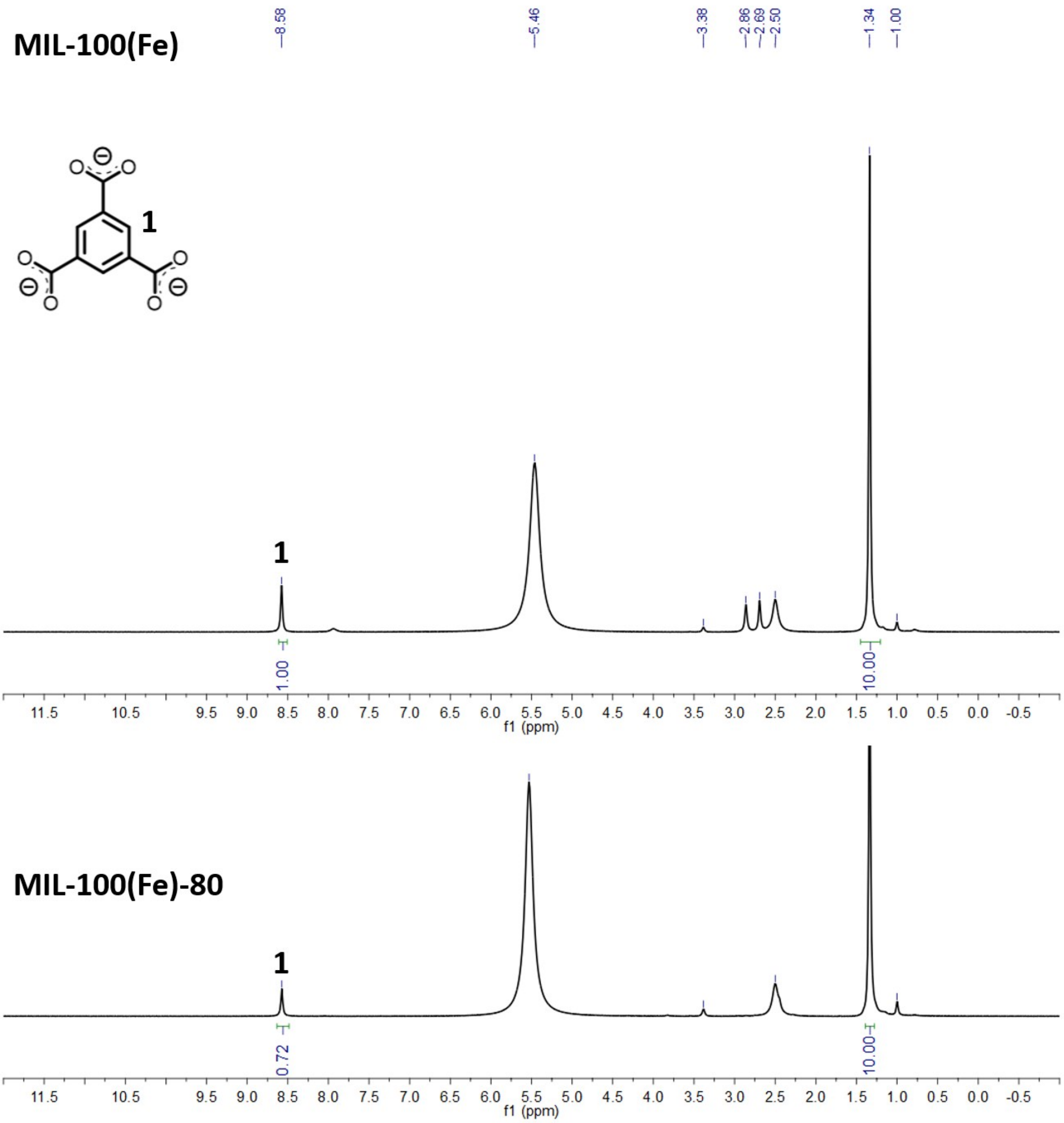

Figure S6. ^{1}H-NMR spectra after digestion with deuterium chloride in DMSO solution of pristine MIL-100(Fe) (a) and MIL-100(Fe)-**80** (b). Cyclohexane (1.34 ppm) was added as an internal standard.

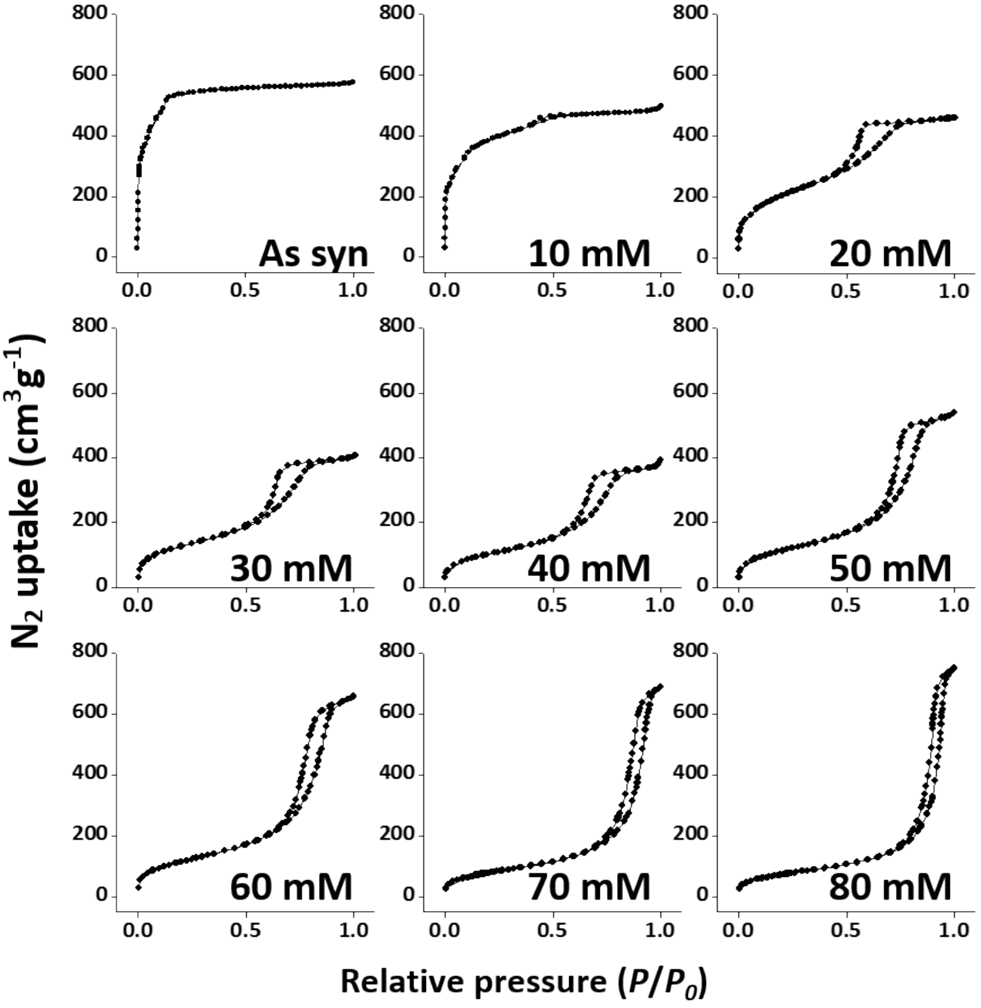

Figure S7. N_2 sorption isotherms of as-prepared MIL-100(Fe) and acid treated MIL-100(Fe) series.

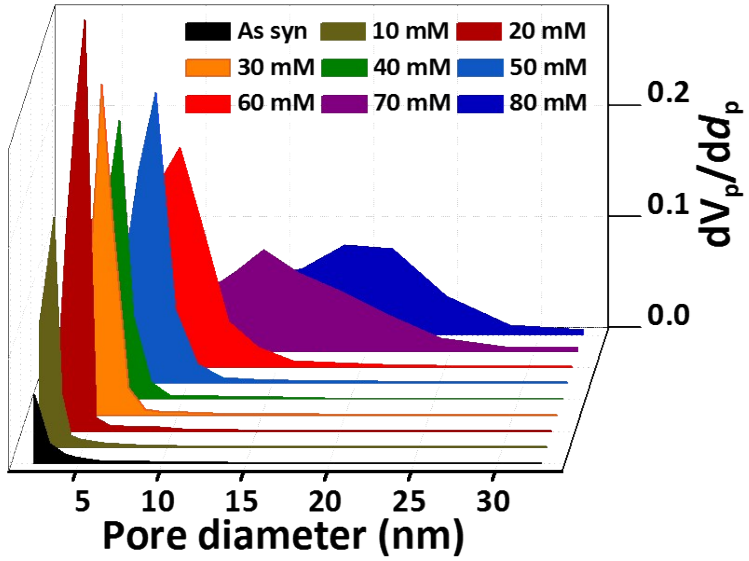

Figure S8. Pore size distribution profiles of as-prepared MIL-100(Fe) and acid treated MIL-100(Fe) series.

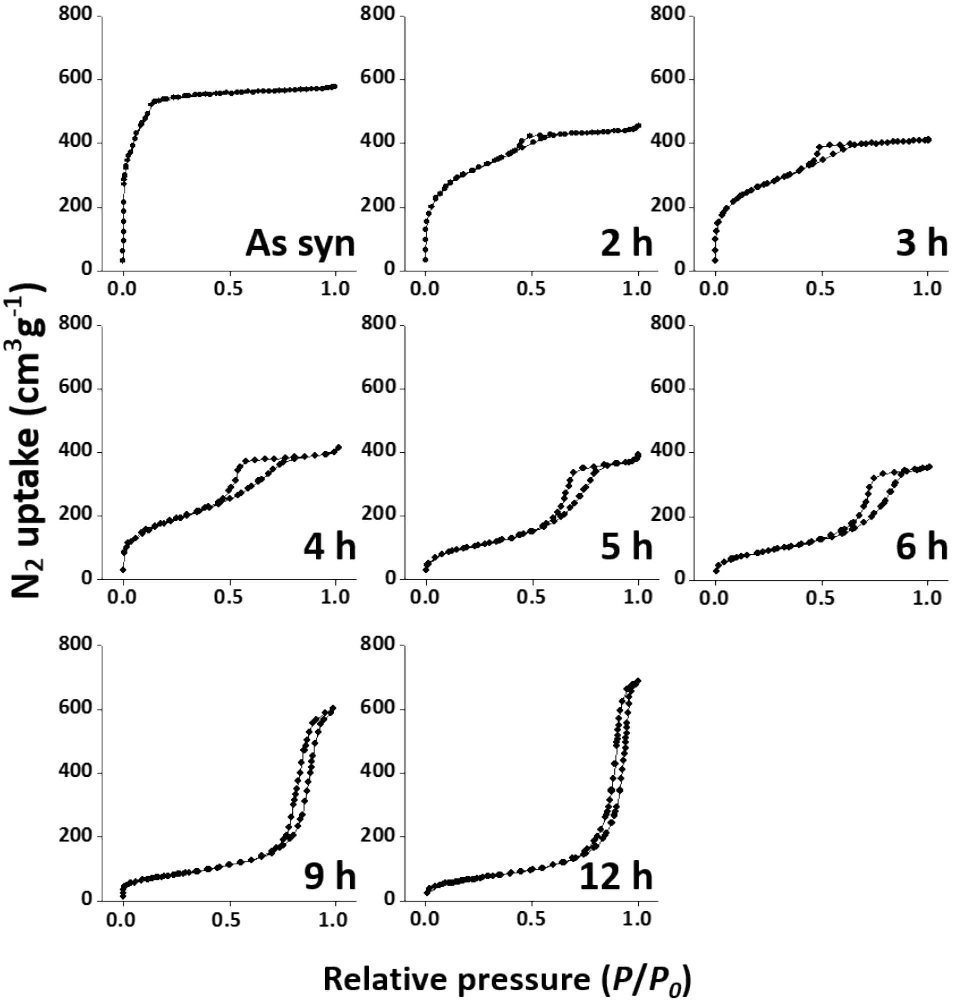

Figure S9. N_2 sorption isotherms of acid treated MIL-100(Fe) for different periods of time (the acid concentration of 40 mM and the temperature of 70 ºC were constant).

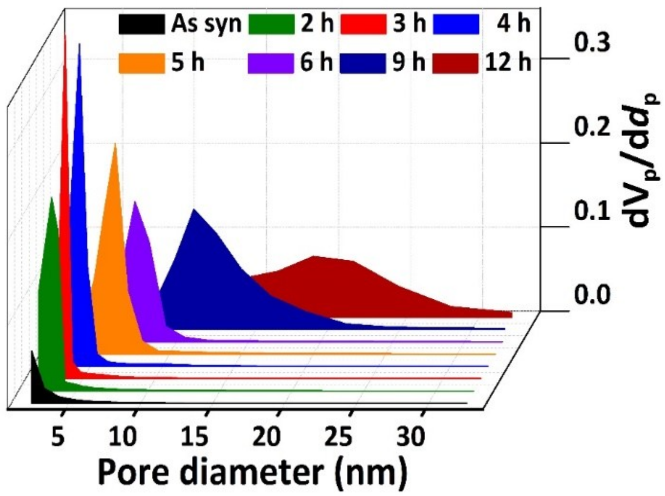

Figure S10. Pore size distribution of acid treated MIL-100(Fe) for different periods of time.

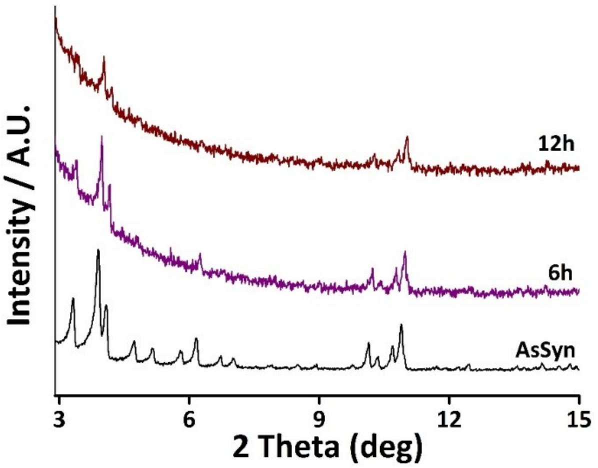

Figure S11. PXRD profiles of as-synthesized MIL-100(Fe) and acid-treated MIL-100(Fe) for 6h and 12h.

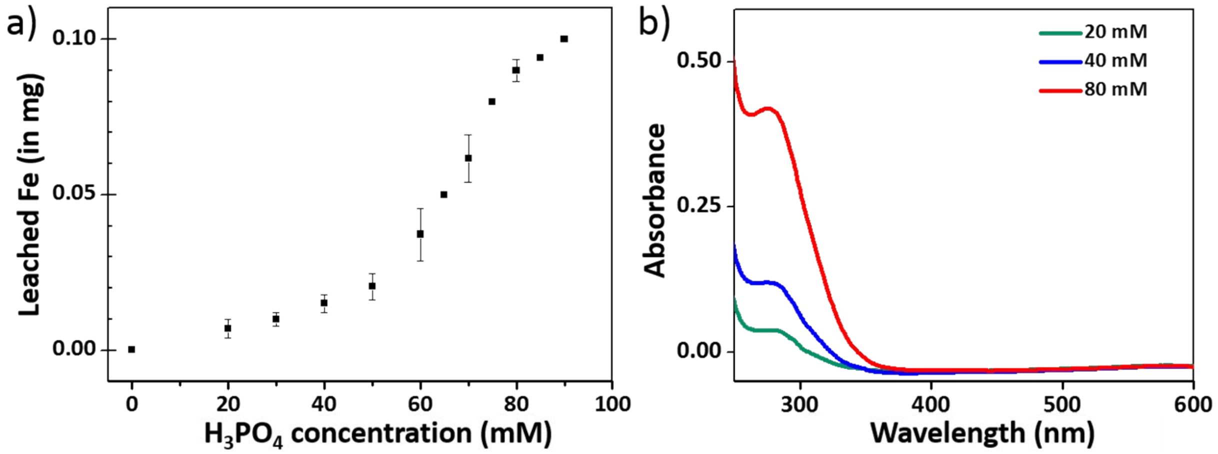

Figure S12. Analyses of the leached Fe^{3+} (a) and BTC ligand (b) during the transformation by ICP-AES and UV-Vis spectroscopy, respectively.

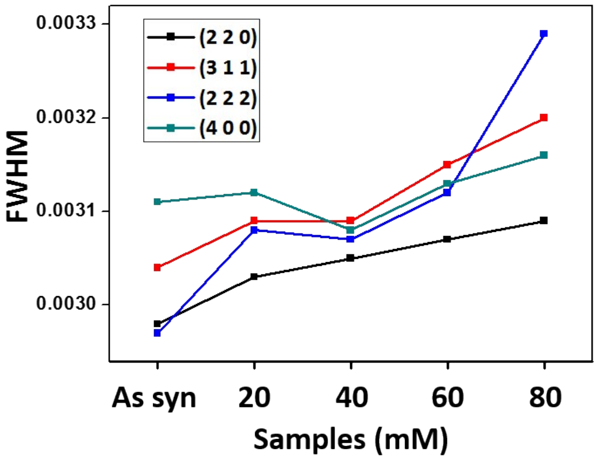

Figure S13. FWHM plots calculated based on the SAXS profiles of as-synthesized MIL-100(Fe) and acid treated MIL-100(Fe) series.

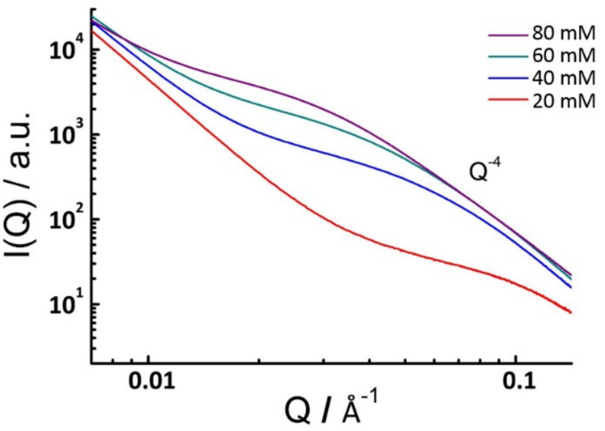

Figure S14. SAXS profiles of acid treated MIL-100(Fe). ($Q < 0.1$ Å^{-1})

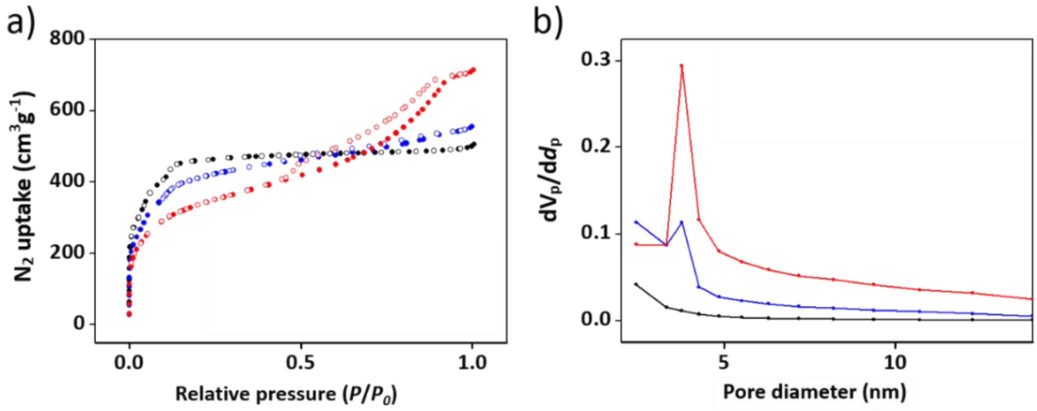

Figure S15. N_2 sorption isotherms (a) and mesopore size distribution (b) of H_2SO_4 treated MIL-100(Fe), (black: pristine, blue: treated by 10 mM H_2SO_4 solution, red: treated by 20 mM H_2SO_4 solution).

Mesoporous transformation procedure of soc-MOF and MIL-88A

soc-MOF and MIL-88A were prepared according to the literature procedures.[3, 4] Dehydrated soc-MOF or MIL-88A crystals (100 mg) were soaked the H_3PO_4 solution where the acidity was adjusted in each case. After the acid etching, the crystals were washed thoroughly with DMF and EtOH 3 times each and dried under vacuum overnight.

Single crystal X-ray diffraction of soc-MOF

Single crystal data of soc-MOF was obtained using a ADSC Quantum 210 CCD diffractometer located in Macromolecular Crystallography Wiggler Beamline 2D in Pohang Accelerator Laboratory (PAL). The diffraction data was collected by using synchrotron radiation (l = 0.7000 Å) with an exposure time of 10 s per frame and a total of 360 frames using the ADSC Q210 ADX program that is rotated by the scan width of 1.00 in the w angle at 100 K. The crystal data was collected to the primitive triclinic lattice system (space group P1) for a full set data, and it was processed and scaled by using HKL3000. The matched Bravais lattice systems were concluded to be the cubic lattice system by auto indexing of the collected diffraction peaks. The crystal structure of soc-MOF was solved by the direct method using the SHELXTL-XS program and refined by full-matrix least-squares calculations using SHELXTL-XL (Ver. 2008) program package. In the structural refinements, the chemical elements were modified using the instruction of DISP (the dispersion and the absorption coefficient of particular elements) at the wavelength of 0.7000 Å

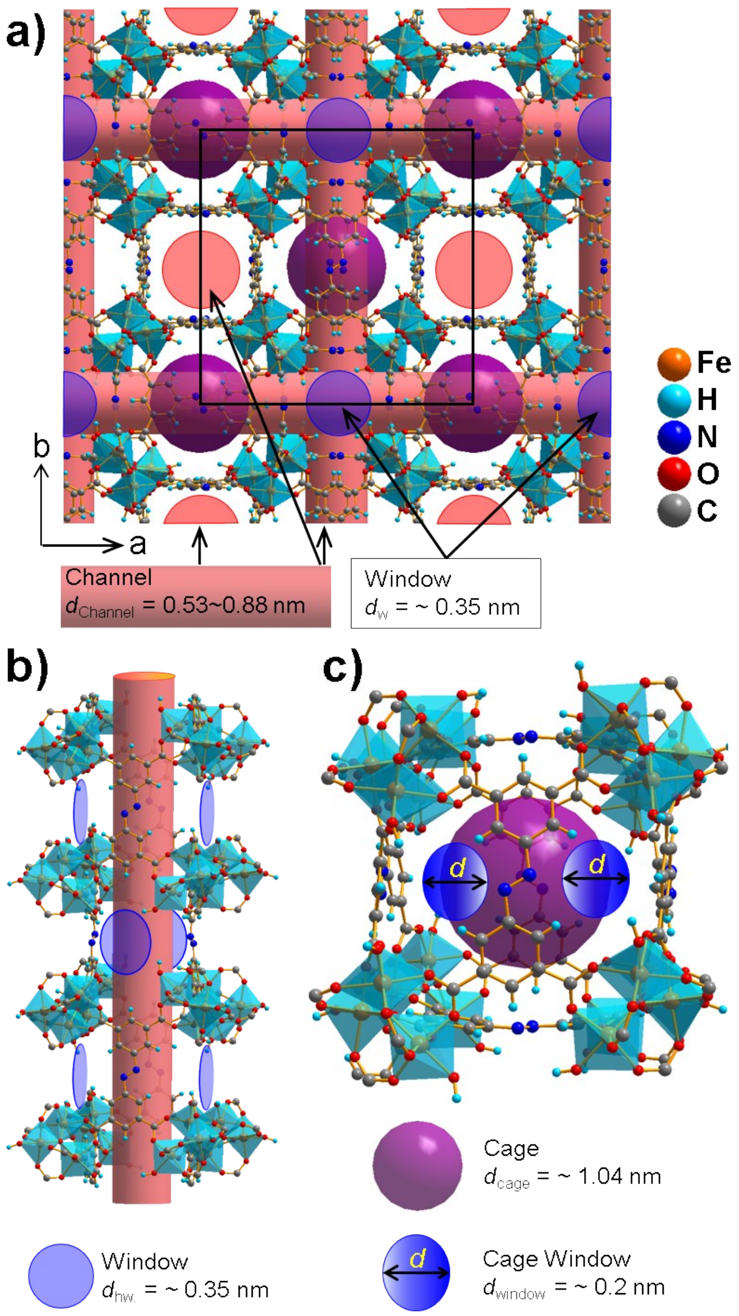

Figure S16. Illustration of channels, cages, and their windows in soc-MOF. a) Unit cell structure; b) Partial structure of large channels and small windows with a diameter of ~0.35 nm; c) Local structure with two kinds of cages.

soc-MOF is a recently reported micro-porous MOF with a space group of P-43n (cubic system) and a unit cell parameter of a = 21.921(3) Å. It contains one kind of cage (purple balls, diameter is around 1.04 nm) and 3D straight channels with shared small windows (diameter = ~0.35 nm, blue circles).[2,5,6] Therefore, we inferred that H_3PO_4 diffused into the large channel, but not through the small window.

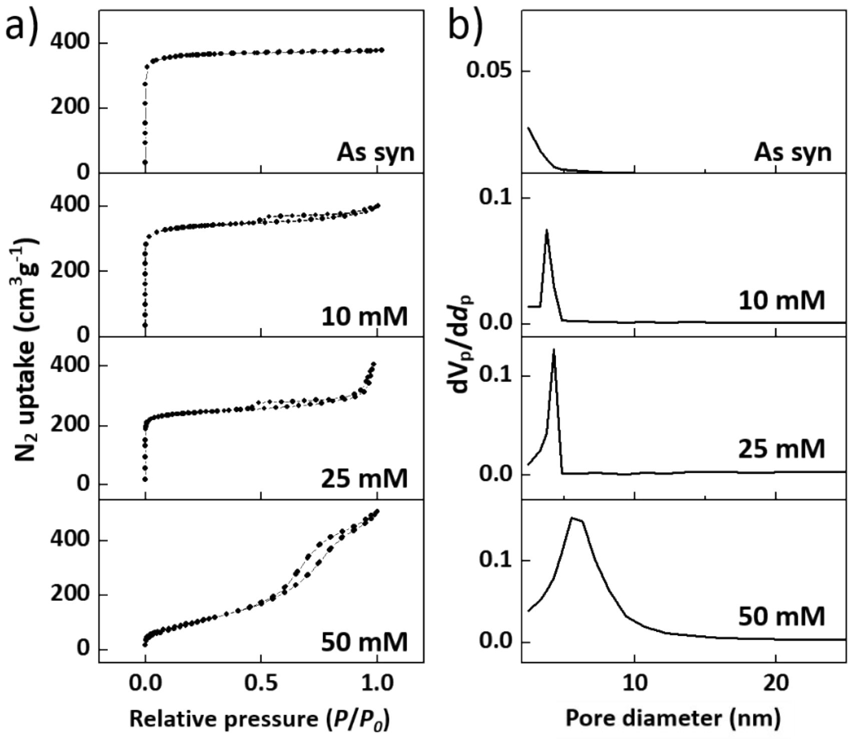

Figure S17. N_2 sorption isotherms (a) and mesopore distribution (b) of pristine soc-MOF and H_3PO_4 treated soc-MOF.

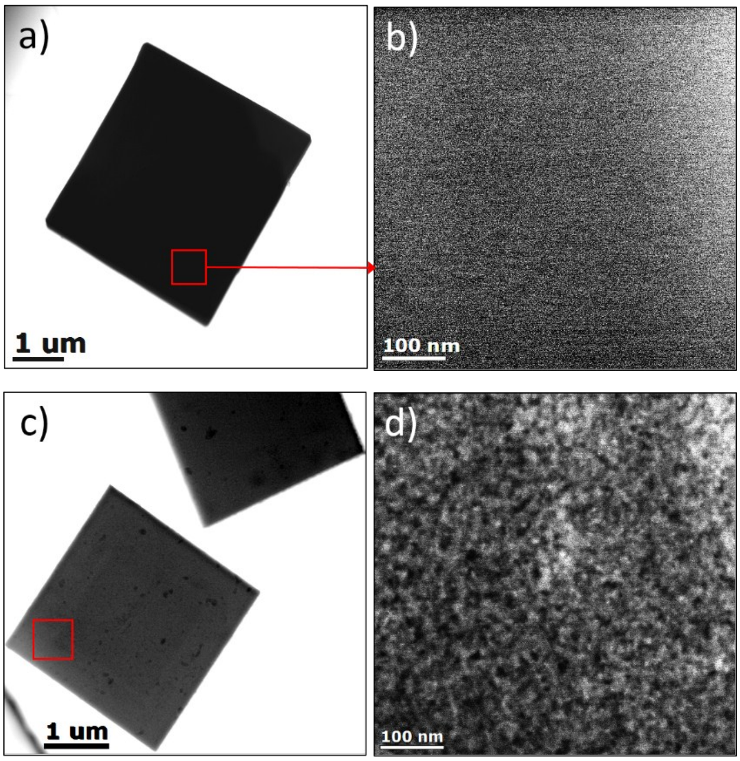

Figure S18. Scanning transmission electron microscopy (STEM) images of pristine soc-MOF (a-b) and H_3PO_4 treated soc-MOF (c-d).

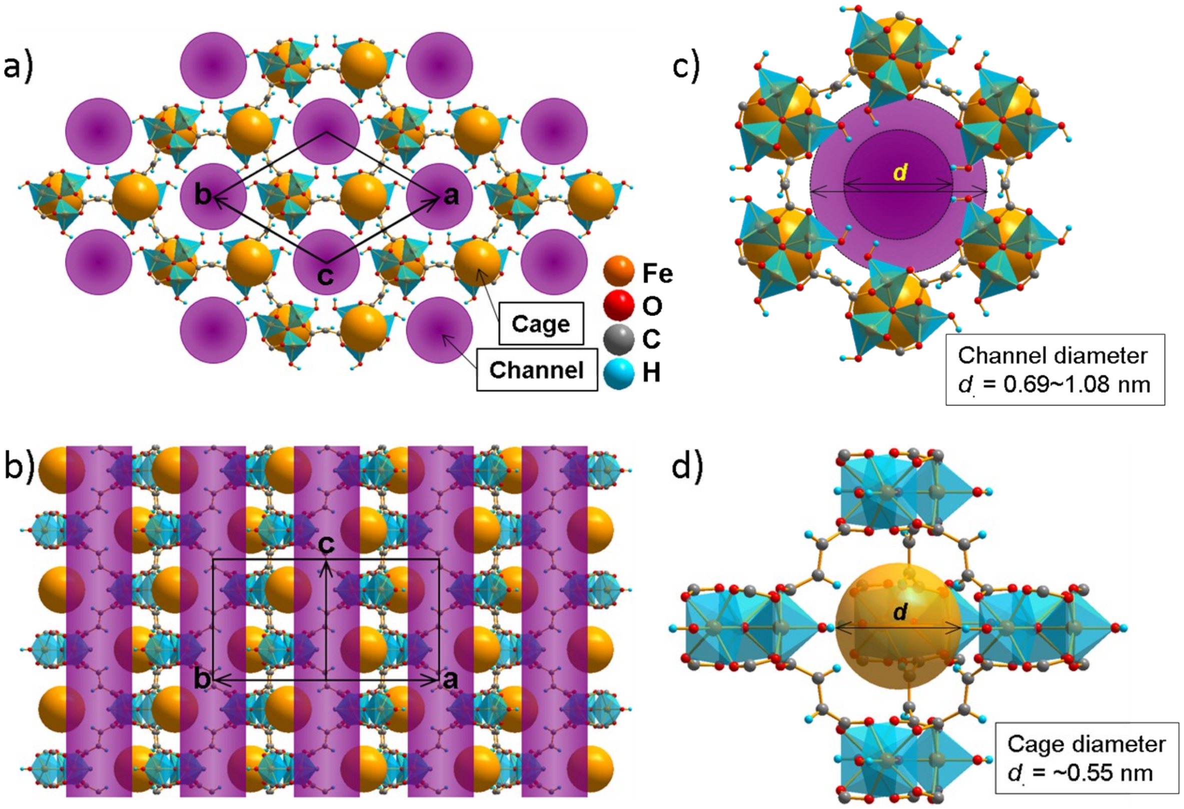

Figure S19. Illustration of channels, cages, and their windows in MIL-88A(Fe). The large channels allow the selective diffusion of H_3PO_4, while the small cages maintain their crystallinity. a) Unit cell structure with cages (orange colour) and channels (purple colour) viewed along c-axis; b) magnified local structure from a; c) side view of the channels with surrounded cages; d) magnified local structure from c.

Porous MIL-88A has a very large, reversible, swelling effect due to its flexible crystalline framework[6], which causes its cell volume to almost double (85%) while fully retaining its open-framework topology. Powder X-ray diffraction data of MIL-88A were collected for sorption characterization of MIL-88-solvents (dry, EtOH, DMF, H_2O) at room temperature and power conditions (40 kV, 30 mA). Unit cell parameters of all MIL-88A's were calculated by the structure-less whole powder pattern fitting (WPPF) in Rietveld analysis (using PDXL-software) (Table S2).

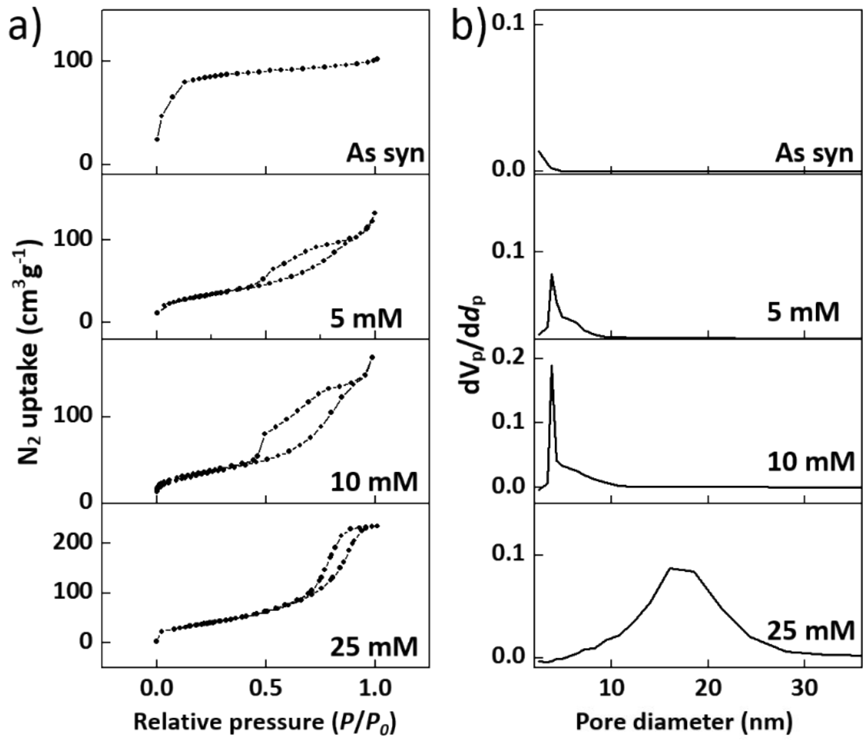

Figure S20. N_2 sorption isotherms (a) and mesopore distribution (b) of pristine MIL-88A and H_3PO_4 treated MIL-88A.

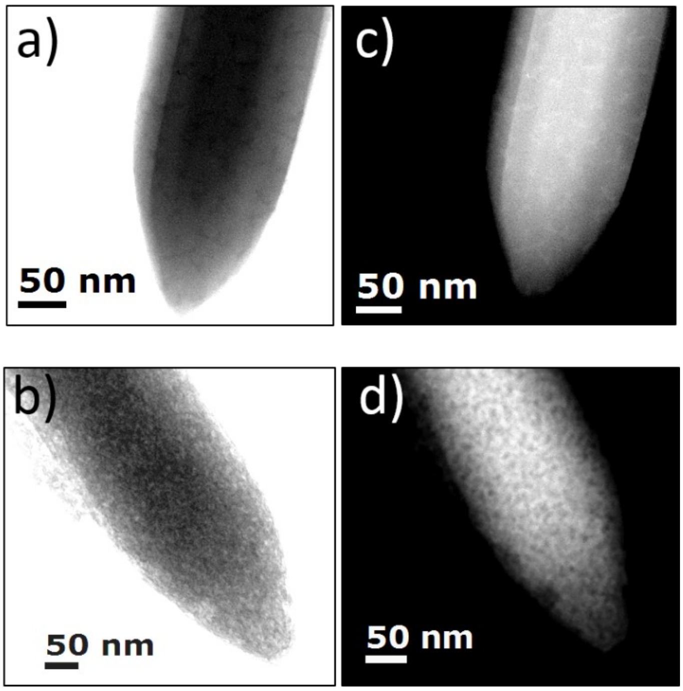

Figure S21. STEM images of pristine MIL-88A (a, c) and H_3PO_4 treated MIL-88A (b, d).

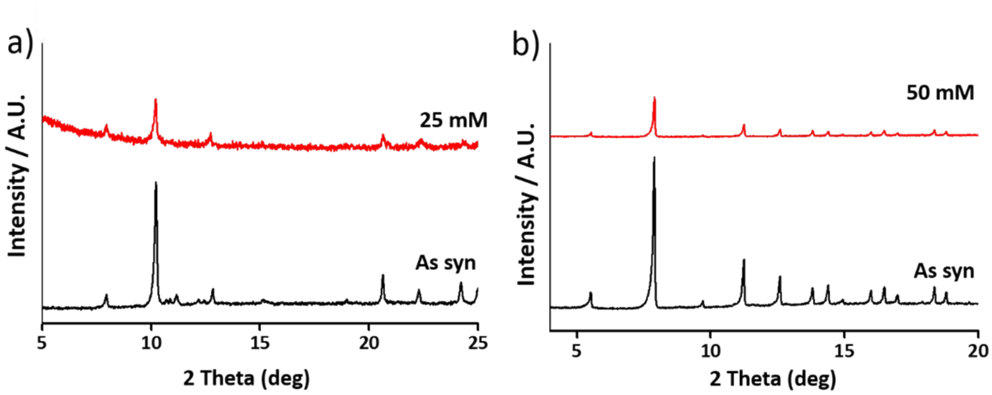

Figure S22. PXRD profiles of pristine (black) and H_3PO_4 treated (red) MIL-88A (a) and soc-MOF (b).

2. Supplementary Tables

Table S1. Pore features of as-synthesized MIL-100(Fe) and acid treated MIL-100(Fe) series.

H_3PO_4 (mmol/L)	${}^{a}S.A._{BET}$ (m^2/g)	${}^{b}V_t$ (cm^3/g)	${}^{c}V_{micro}$ (cm^3/g)	${}^{d}V_{meso}$ (cm^3/g)	${}^{e}D_{meso}$ (nm)	${}^{c}V_{micro}/{}^{d}V_{meso}$
**0**	2020	0.8918	0.8561	0.1059	2.43	8.084
**10**	1350	0.7647	0.6848	0.3098	3.32	2.210
**20**	730	0.7113	0.4161	0.5986	4.82	0.695
**30**	445	0.6216	0.2618	0.5809	5.47	0.451
**40**	359	0.5981	0.2118	0.5657	6.23	0.374
**50**	404	0.8287	0.2341	0.8077	8.1	0.290
**60**	398	1.0162	0.2422	0.9954	9.25	0.243
**70**	305	1.0598	0.1653	1.0386	13.88	0.159
**80**	271	1.1556	0.1518	1.1509	18.44	0.134

${}^{a}S.A.BET$ is the Brunauer-Emmett-Teller (BET) surface area.
${}^{b}V_t$ is the total pore volume calculated by BET-analysis.
${}^{c}V_{micro}$ is the specific micropore volume calculated by HK-analysis.
${}^{d}V_{meso}$ is the specific mesopore volume calculated by BJH-analysis.
${}^{e}D_{meso}$ is the pore diameter calculated by BJH-analysis.

Table S2. Cell Parameters of the MIL-88A under varied conditions

condition	a (Å)	c (Å)	volume (Å^3)	
Dried at RT	11.247(2)	14.648(5)	1604.6(6)	This work
DMF/H_2O	12.708(2)	13.684(4)	1913.9(6)	
EtOH/H_2O	13.433(6)	13.084(7)	2044.7(9)	
H_2O	13.652(9)	12.691(9)	2048.3(9)	

dried at 423 K	9.26	15.31	1135	Ref. [7]
as-syn. (dried at RT)	11.18	14.59	1580	
C_4H_9OH	12.46	13.70	1840	
C_2H_5OH	13.07	13.30	1970	
CH_3OH	13.78	12.69	2090	
H_2O	13.87	12.66	2110	

3. References

[1] P. Horcajada, S. Surble, C. Serre, D. Y. Hong, Y. K. Seo, J. S. Chang, J. M. Greneche, I. Margiolaki and G. Férey, *Chem. Commun.*, 2007, **27**, 2820-2822.

[2] Y. A. Fadeeva, L. P. Safonova and I. Persson, *Phy. Chem. Chem. Phy.*, 2010, **12**, 8977-8984.

[3] M. Pang, A. J. Cairns, Y. Liu, Y. Belmabkhout, H. C. Zeng and M. Eddaoudi, *J. Am. Chem. Soc.*, 2013, **135**, 10234-10237.

[4] L. Wang, Y. Zhang, X. Li, Y. Xie, J. He, J. Yu and Y. Song, *Sci. Rep.*, 2015, **5**, 14341-14352.

[5] D. Feng, K. Wang, Z. Wei, Y. P. Chen, C. M. Simon, R. K. Arvapally, R. L. Martin, M. Bosch, T. F. Liu, S. Fordham, D. Yuan, M. A. Omary, M. Haranczyk, B. Smit and H. C. Zhou, *Nat. Commun.*, 2014, **5**, 5713-5720.

[6] H. Chevreau, A. Permyakova, F. P. Fabry, C. Livage, F. Ragon, A. G. Marquez, T. Devic, N. Steunou, C. Serre and P. Horcajada, *CrystEngComm.*, 2016, **18**, 4094-4101.

[7] C. M. Draznieks, C. Serre, S. Surblé, N. Audebrand and G. Férey, *J. Am. Chem. Soc.*, 2005, **127**, 16273-16278.
